# The Impact of Illegal Waste Sites on the Transmission of Zoonotic Cutaneous Leishmaniasis in Central Tunisia

**DOI:** 10.3390/ijerph18010066

**Published:** 2020-12-24

**Authors:** Ifhem Chelbi, Olfa Mathlouthi, Sami Zhioua, Wasfi Fares, Anis Boujaama, Saifedine Cherni, Walid Barhoumi, Khalil Dachraoui, Mohamed Derbali, Mohamed Abbass, Elyes Zhioua

**Affiliations:** 1 Laboratory of Vector Ecology, Pasteur Institute of Tunis, 1002 Tunis, Tunisia; ifhemc2001@yahoo.fr (I.C.); olfa85mathlouthi@yahoo.fr (O.M.); fwasfi@yahoo.fr (W.F.); saifcherni@yahoo.com (S.C.); walidbarhoumi.ipt2009@yahoo.fr (W.B.); khalil.dachraoui@yahoo.com (K.D.); derbali.hiba@gmail.com (M.D.); m.abbas9900@gmail.com (M.A.); 2 Laboratory of Biostatistics, Pasteur Institute of Tunis, 1002 Tunis, Tunisia; sami.zhioua2@gmail.com; 3 National Institute of Statistics, 1002 Tunis, Tunisia; anis.boujaama@gmail.com

**Keywords:** illegal waste sites, sandflies, rodents, leishmaniasis, waste management

## Abstract

Illegal waste disposal represents a risk health factor for vector-borne diseases by providing shelter for rodents and their ectoparasites. The presence of the *Phlebotomus papatasi* vector of *Leishmania major*, an etiologic agent of zoonotic cutaneous leishmaniasis (ZCL), was assessed at illegal waste sites located at the vicinity of villages in endemic areas of Central Tunisia. The study was performed over a two-year period over three nights from July to September 2017, and over three nights in September 2018. Household waste is deposited illegally forming dumpsites at the vicinity of each village and contains several rodent burrows of *Psammomys obesus*, the main reservoir host of *L. major*. Sandflies were collected from rodent burrows in the natural environment and in dumpsites using sticky traps and were identified at species level. Female sandflies were tested for the presence of *L. major* by PCR. Our entomological survey showed that *Phlebotomus papatasi* is the most abundant sandfly species associated with rodent burrows in these waste sites. The densities of *P. papatasi* in dumpsites are significantly higher compared to the natural environment. The minimum infection rate of *P. papatasi* with *L. major* in these illegal waste sites is not significantly different compared to the natural environment. Considering the short flight range of *P. papatasi*, increases in its densities, associated with burrows of *P. obesus* in illegal waste sites located at the edge of villages, expands the overlap of infected ZCL vectors with communities. Thus, illegal waste sites pose a high risk of spreading ZCL to neighboring home ranges. Waste management is an environmentally friendly method of controlling sandfly populations and should be included in an integrated management program for controlling ZCL in endemic countries.

## 1. Introduction

Zoonotic cutaneous leishmaniasis (ZCL) is a neglected tropical disease that affects thousands of people annually in the endemic areas of Central and Southern Tunisia [1,2]. This disease is caused by the parasite *Leishmania major*, and is transmitted by the sandfly *Phlebotomus papatasi* [3,4]. In this region, the fat sand rat, *Psammomys obesus*, and the desert jird *Meriones shawi*, are the principal reservoir hosts of *L. major* [5,6,7,8,9,10]. Although ZCL is generally not fatal, the lesions produced may cause substantial disfigurement and severe distress to infected individuals with lifelong psychological and social consequences [11]. There is no ZCL vaccine available and treatment is based on chemotherapy. In addition, programs concerning vector and reservoir host control are currently absent for the prevention of this neglected tropical disease.

Following the construction of the dam of Sidi Saad in Central Tunisia and the resettlement of non-immune human populations, a major outbreak of ZCL occurred in 1982–1983 [12]. A total of 15,897 ZCL cases were reported from 1999 to 2004 with an average annual incidence rate of 669.7 per 100,000 inhabitants in the governorate of Sidi Bouzid [13]. About 63.4% of the cases were from rural areas, which account for 71% of the total population of the governorate of Sidi Bouzid (395,506 inhabitants).

Rural areas located in Central Tunisia are characterized by poor housing conditions and unsuitable waste management, where villages are surrounded by chenopod fields that consist of salt flats of halophytic vegetation, predominantly populated by plants of the family Chenopodiaceae: *Salsola tetrandra*, *Suaeda fruticosa*, and *Arthrocnemum glaucum*. These salt flats are used also as grazing areas for livestock [14].

These chenopod fields are also the natural habitats of *P. obesus*, the main reservoir host of *L. major* [14]. In the absence of total waste management, household waste made of materials such as rubber, plastic, animal dung, and earthen and organic matter is deposited at the vicinity of villages, creating dumpsites. Illegal waste is deposited on the outskirts of villages and can sustain populations of *P. obesus* [15]. As a general assumption, adult sandflies in villages come from breeding and resting sites located on the surrounding land [16]. We hypothesized that illegal waste sites deposited at the edge of villages impact the breeding of *P. papatasi* and subsequently increase the transmission risk of ZCL.

## 2. Materials and Methods

**Figure 1:**
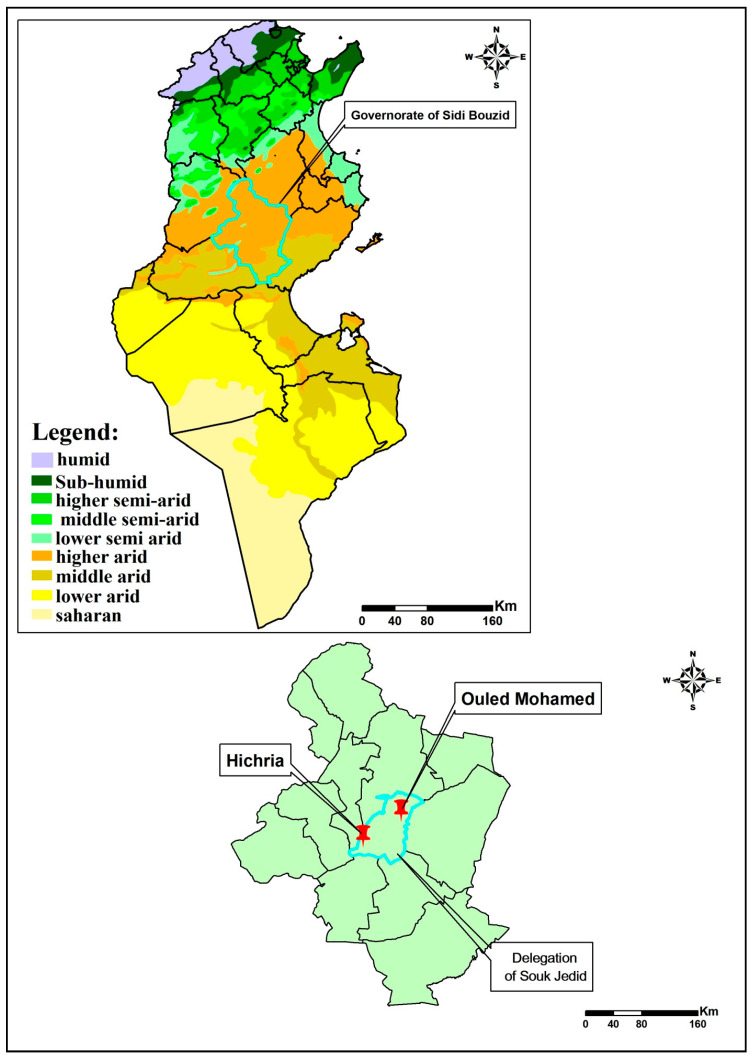
Bioclimatic map of Tunisia showing sampling sites in the governorate of Sidi Bouzid.

The study was performed in the villages of Hichria (34°50′ N, 9°28′ E) and Ouled Mohamed (34°53′ N, 9°35′ E), located in the governorate of Sidi Bouzid (Figure 1), a highly endemic area with multiple foci of ZCL [1,13]. Hichria and Ouled Mohamed are 15 km away from one another and belong to the delegation of Souk Jedid.

**Figure 2:**
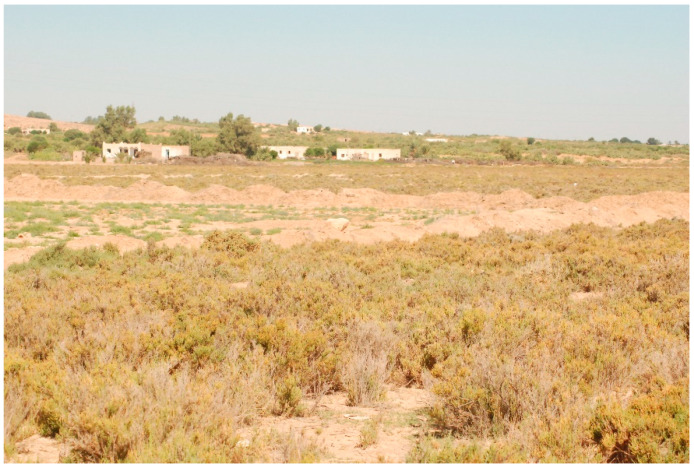
Chenopod fields surrounding villages.

**Figure 3:**
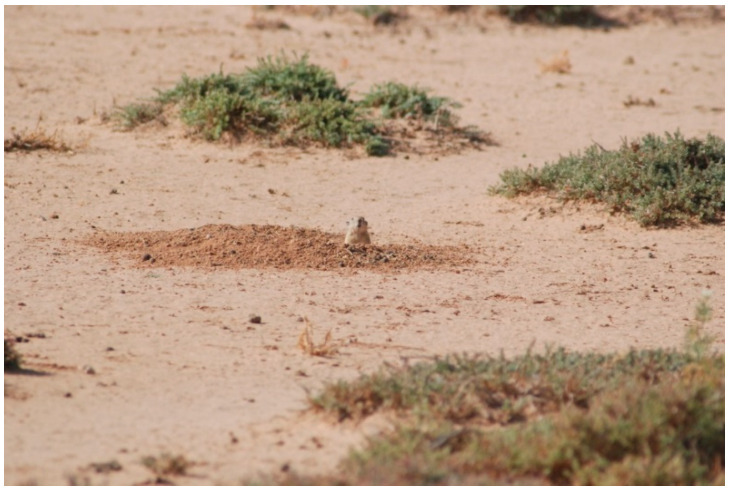
*Psammomys obesus* in its natural habitat (chenopod field; A).

**Figure 4:**
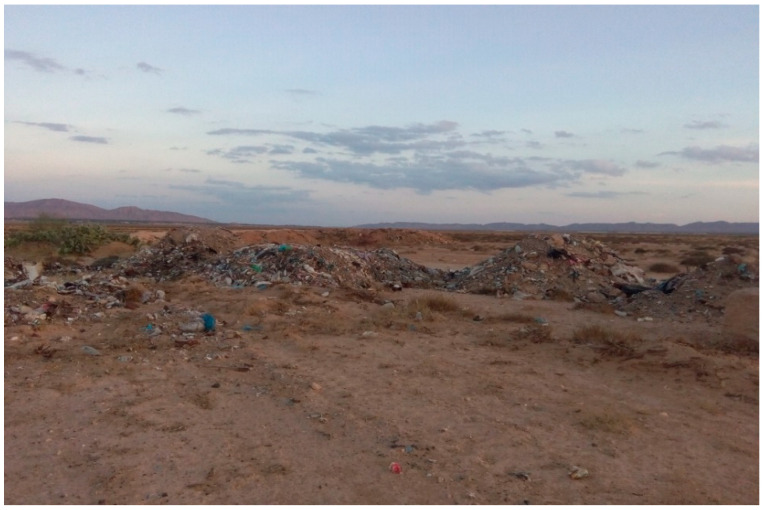
Dumpsites made of household waste adjacent to chenopod fields.

Both villages are surrounded by non-agricultural fields made of chenopods (Figure 2), which are the natural habitat of *P. obesus* (Figure 3). Household waste is deposited illegally, forming dumpsites at the outskirts of each village and containing several burrows of *P. obesus* [15] (Figure 4). Thus, around each village, a natural site adjacent to a waste disposal site is considered in this study.

Both dumpsites made of household waste and chenopod fields surrounding the villages sustain sand rat colonies [15]. In Tunisia, the phenology of *P. papatasi* is characterized by two main peaks of activity: one in June-July and a second, larger peak in September-October [1]. The collection of sandflies was performed in 2017 over three nights (1 night in July and 2 nights in September). The same entomological survey was performed over three nights in September 2018.

Sandflies were collected using sticky traps. Each trap consists of 13 white sheets of paper (20 cm × 20 cm) soaked in castor oil, yielding a total surface of 1 m^2^. The density is reported as the number of a sandfly species per 1 m^2^ of sticky traps [1]. Three m^2^ of sticky traps, corresponding to 3 replicates, were placed inside active rodent burrows from dusk to dawn at each of the four sites. Active rodent burrows are characterized by the presence of Chenopodiaceae fragments and feces at their entries [14]. Taking into account the number of sites (*n* = 4) and the number of rodent burrows per site (*n* = 39), the total number of trap nights was 468. Collected sandflies were identified individually at species level according to morphological characteristics [17]. Sandflies were then pooled based on collection date with a maximum of 30 unfed females per pool and stored at −80° until use.

Female sandflies in pools of up to a maximum of 30 specimens per pool were homogenized in 200 µL of phosphate-buffered saline (PBS) solution through high-speed shaking using the automated Tissue Lyser LT (Qiagen, Germany) with glass beads. The mixture was clarified by centrifugation at 6000× *g* for 2 min for use in DNA extraction with a Qiagene DNA Mini Kit (Qiagen, Germany) according to the manufacturer’s instructions. *L. major, L. infantum* and *L. tropica* DNA previously extracted from parasite cultures were used as positive controls. DNA extracted from sandfly pools was screened for the presence of *Leishmania* DNA (as a proxy for *Leishmania* infection) using a nested PCR-based schizodeme method targeting the partially conserved region of the kinetoplast minicircle DNA, as described previously [18]. The method enabled *Leishmania* species discrimination on the basis of PCR amplicon size, where *L. tropica* generated a 750-bp product, *L. infantum* produces a 680-bp product, whereas the product size of *L. major* was 560 bps. For each DNA sample extracted from pools of sandflies, an initial amplification step was performed using the Taq DNA recombinant polymerase kit (Invitrogen) in a 50 µL reaction containing 5 µL 10X buffer, 3 µL MgCl2 (50 mM), 2 µL dNTP mix (10 mM), 1 µL of each reverse and forward primer (CSB2xF/CSB1xR (10 µM)), 0.5 µL Taq and 10 µL of total extracted DNA. The nested PCR was carried out in a 50 µL reaction containing 3 µL of the initial PCR and 47 µL of a mixture containing 5 µL 10X buffer, 3 µL MgCl2 (50 mM), 2 µL dNTP mix (10 mM), 1 µL of each reverse and forward internal primer (13Z/LIR (10 µM)) and 0.5 µL of Taq DNA polymerase (Invitrogen). Optimized cycling conditions for the first and second PCR step were performed as follows: 94 °C for 5 min followed by 35 cycles, repeating denaturation at 94 °C for 30 s, annealing at 55 °C for 60 s and elongation at 72 °C for 90 s, and an extension step at 72 °C for 10 min. Cross-contamination was monitored by negative controls for sample extraction and PCR solutions. Amplification products of the nested PCR were confirmed by electrophoresis in ethidium bromide-stained 2% agarose gel. Positive PCR product sizes were estimated according to 100 bp molecular weight (Invitrogen) to identify sandfly-associated *Leishmania* species. In this study, the infection of *P. papatasi* by *L. major* is reported using the minimum infection rate (MIR) which is calculated: ([number of positive pools/total number of tested sandflies] × 100) [19].

We conducted an overall analysis to evaluate whether densities of *P. papatasi* are higher in dumpsites compared to chenopod fields. The densities of sandflies and the MIRs were analyzed using a non-normal distribution approach. A Wilcoxon rank sum test was used to study the null hypothesis with no significant difference between chenopod fields and dumpsites in terms of sandfly densities and in term of MIRs. *p* values were considered significant at 0.05. Statistics were assessed using R 3.6.0.

## 3. Results

A total of 666 and 528 sandflies were collected from Hichria and Ouled Mohamed during 2017, respectively. Morphological identification of sand files showed that *P. papatasi* was the most abundant species in Hichria and in Ouled Mohamed (Table 1). A total of 361 and 291 sandflies were collected from Hichria and Ouled Mohamed during 2018, respectively. Morphological identification of sand files showed that *P. papatasi* was the most abundant species in both villages (Table 1). Other sandflies were identified such as *Phlebotomus perniciosus* and *Sergentomyia minuta,* but their abundances are low compared to *P. papatasi* (Table 1).

**Figure 5:**
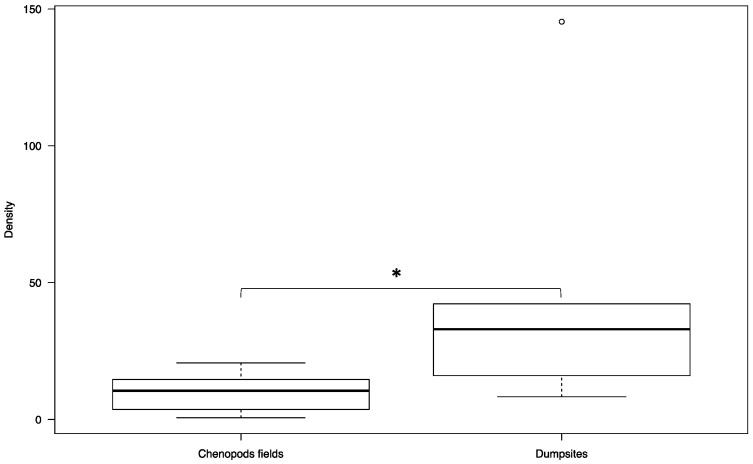
Overall densities of *Phlebotomus papatasi* in the chenopod fields and in the dumpsites adjacent to the village of Hichria. (The thick black line represents the median. Lower and upper lines represent the first and third quartile, respectively. Whiskers show the minimum and maximum values. Outliers are represented by circles. Significance: * < 0.05.

**Figure 6:**
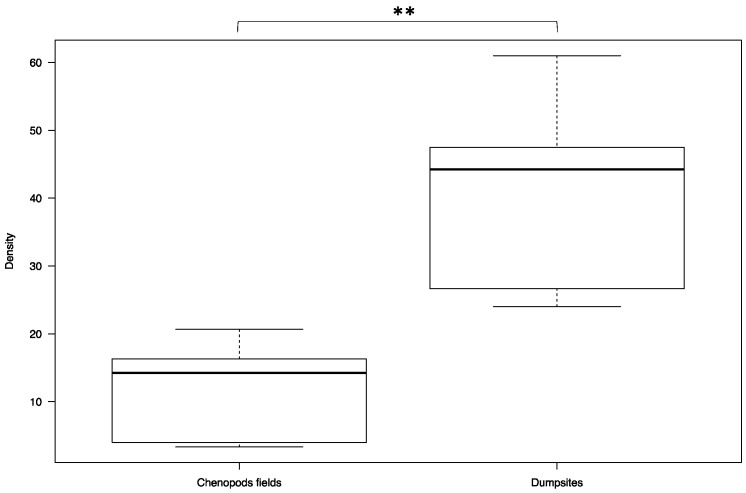
Overall densities of *Phlebotomus papatasi* in the chenopod fields and in the dumpsites adjacent to the village of Ouled Mohamed. (The thick black line represents the median. Lower and upper lines represent the first and third quartile, respectively. Whiskers show the minimum and maximum values. Significance: ** < 0.01.)

In Hichria, the overall sandfly densities in the dumpsites and in the chenopod fields were 46.33 ± 8.39 and 10.10 ± 1.22, respectively. The overall density in the dumpsites is significantly higher compared to the density in the chenopod fields (W = 5, *p*-value = 0.04113) (Figure 5). In Ouled Mohamed, the overall sandfly densities in the dumpsites and in the chenopod fields were 41.28 ± 2.33 and 12.14 ± 1.18, respectively. Similar to the village of Hichria, the overall density in the dumpsites is significantly higher compared to the density in the chenopod fields (W = 0, *p*-value = 0.002165) (Figure 6).

In 2017, a total of 177 and 29 *P. papatasi* females collected from the waste disposal site and from the chenopod fields surrounding the village of Hichria, respectively, were tested for *Leishmania* infection by nested PCR (Table 2). The MIRs in the waste disposal site and in the chenopod fields surrounding the village of Hichria were 2.25, and 3.44, respectively. A total of 125 and 22 *P. papatasi* females collected in 2017 from the waste disposal site and from the chenopod fields surrounding the village of Ouled Mouhamed, respectively, were tested for *Leishmania* infection by nested PCR (Table 2). The MIRs in the waste disposal site and in the chenopod fields surrounding the village of Ouled Mohamed were 2.44, and 4.54, respectively. Only *L. major* was detected in the studied areas according the PCR product size during the 2017 season.

**Figure 7:**
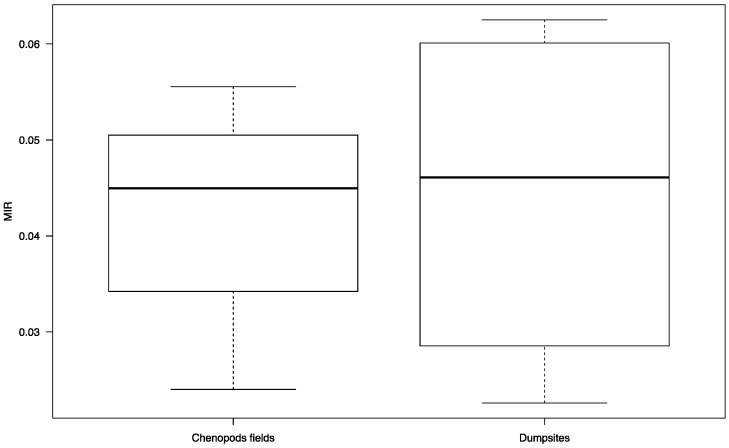
The minimum infection rates of *P. papatasi* with *L. major* in dumpsites (*n* = 4) and chenopod fields (*n* = 4). (The thick black line represents the median. Lower and upper lines represent the first and third quartile, respectively. Whiskers show the minimum and maximum values.

In 2018, a total of 52 and 16 *P. papatasi* females from the waste disposal site and from the chenopod fields surrounding the village of Hichria, respectively, were tested for *Leishmania* infection by nested PCR. The MIRs in the waste disposal site and in the chenopod fields surrounding the village of Hichria were 5.76 and 6.25, respectively (Table 2). A total of 90 and 18 *P. papatasi* females from the waste disposal site and from the chenopod fields surrounding the village of Ouled Mohamed, respectively, were tested for *Leishmania* infection by nested PCR (Table 2). The MIRs in the waste disposal site and in the chenopod fields surrounding the village of Ouled Mohamed were 4.44 and 5.55, respectively (Table 2). Similar to the 2017 season, only *L. major* was detected in studied areas according the PCR product size during the 2018 season. Overall, the MIRs in the dumpsites and in the chenopod fields were 4.43 ± 0.32 and 4.24 ± 0.22, respectively. No significant difference was found between the MIRs in both sites (W = 7, *p*-value = 0.8857) (Figure 7).

## 4. Discussion

In ZCL endemic foci, waste sites rich in organic waste, rubber, plastic, animal dung, and earthen matter deposited illegally at the edge of villages and close to chenopod fields form dumpsites that attract rodents, particularly *P. obesus*. These dumpsites are of major interest in relation to *P. obesus* as they provide shelter from heavy rainfall and subsequent drowning, as well as proximity to chenopods, which represent their main food source (14). It is of major epidemiological importance to point out that these illegal waste sites are within 1 km of villages. Our entomological survey, performed over a two-year period, showed that illegal waste sites sustained a large population of *P. papatasi*. In these waste sites, active rodent burrows have moderate, stable temperatures, elevated humidity, and are rich in organic matter, which creates a suitable microclimate for the immature and adult stages of *P. papatasi* [10]. Adult *P. papatasi* females utilize *P. obesus* as their primary blood meal source, and the rodent feces, plant debris, and organic waste that accumulate in these burrows are the main food sources for sandfly larvae, and subsequently contribute significantly to sustaining a large population of *P. papatasi*, characterized by high densities. Therefore, a significantly higher density of *P. papatasi* in waste sites compared to natural biotopes is expected. This could be explained by the high abundance of *P. obesus* in the waste sites compared to the chenopod fields around villages [20]. This hypothesis deserves further investigation.

A high MIR in *P. papatasi* females with *L. major* was found in the waste sites. However, no significant difference was observed between the MIRs in the dumpsites and in the chenopod fields. A previous study performed by our group showed that the infection prevalence values for *P. obesus* infected with *L. major* in the same waste sites in Hichria and Ouled Mohamed were 33% and 60%, respectively [15]. The infection prevalence of *P. obesus* with *L. major* captured from the chenopod fields in Ouled Mohamed was reportedly 41% [9]. Similar to the chenopod fields, *P. obesus* is the main source of infection of *P. papatasi* in the waste sites. Since the potential risk of direct transmission of infectious diseases by any kind of solid waste depends on the presence of an infectious agent, its viability in solid waste, and the presence of a susceptible host [21], our results provided strong evidence that illegal waste sites represent a high risk factor of ZCL for the surrounding communities.

It is of major epidemiological importance to point out that the governorate of Sidi Bouzid has the highest ZCL prevalence nationwide [2,22]. In endemic rural areas located in the center of the governorate of Sidi Bouzid, the annual average incidence rate was 502.9 cases per 100,000 inhabitants [22]. Ouled Mohamed has the highest annual incidence of ZCL, with 3822.8 cases per 100,000, followed by Hichria with 1660.4 cases per 100,000 inhabitants [22]. Considering that the flight range of *P. papatasi* is around 0.75 km [23], increases in the densities of *P. papatasi* in illegal waste sites located at the edge of villages expand the overlap of infected ZCL vectors with communities, leading to a high incidence of ZCL. This hypothesis is corroborated by the fact that the distance between the source of the epidemic and the dwellings with human cases in endemic areas of Central Tunisia required to reach an epidemic of ZCL was estimated at approximately 1 km [24]. Thus, illegal waste sites pose a high risk of spreading ZCL to neighboring home ranges. Due to the occupation of land near these dumpsites, at the edge of villages and close to chenopod fields, the natural habitat of *P. obesus* may have established a direct link between the sylvatic and peri-urban cycles of ZCL.

Several studies showed the impact of illegal waste sites on vector-borne diseases such as dengue [25], rodent-borne diseases such leptospirosis [26], toxoplasmosis [27], and zoonotic viruses [28]. However, few studies linking illegal waste and leishmaniasis have been performed. The risk of visceral leishmaniasis in South America increased with the absence of regular trash collection [29]. *Phlebotomus argentipes*, a vector of visceral leishmaniasis in the Indian subcontinent, breeds in the trash [30]. Our findings showed higher densities of *P. papatasi* in illegal waste sites compared to the natural environment. In addition, The MIRs observed in the natural sites and the dumpsites are similar. Thus, the results of our study provided strong evidence that illegal waste sites significantly increase the risk of transmission of ZCL. Therefore, improved waste management is essential in the prevention and the control of ZCL in endemic countries. Unsuitable waste management at the edge of villages located in ZCL endemic areas is providing ideal conditions for breeding and resting sites for *P. papatasi* populations and subsequently increasing the risk of ZCL. Similar to several neglected tropical diseases, such as trachoma, schistosomiasis, soil-transmitted helminthiasis, and Guinea worm, the prevention and control of ZCL also depend on the availability of improved water, sanitation, and hygiene (WASH) in ZCL endemic rural communities [31].

## 5. Conclusions

Based on our entomological findings, we concluded that waste management through the education of communities to resist trash accumulation at the vicinity of villages in order to reduce the risk of ZCL transmission should be included in an integrated vector management program.

## Figures and Tables

**Table 1:** Sandfly fauna collected in Hichria and in Ouled Mohamed in 2017 and 2018.

Years	2017			2018		
Sites	Hichria		Ouled Mohamed	Hichria		Ouled Mohamed
Total numbers of sandflies	666		528	361		291
Genus	*Phlebotomus*		*Phlebotomus*	*Phlebotomus*	*Segentomyia*	*Phlebotomus*
Species	*papatasi*	*perniciosus*	*papatasi*	*papatasi*	*minuta*	*papatasi*
Sex (F/M)	205/453	3/5	148/380	72/285	0/4	108/183
Abundance (%)	98.8	1.2	100	98.2	1.2	100

**Table 2:** Minimum infection rates of *Phlebotomus papatasi* with *Leishmania major* in Hichria and in Ouled Mohamed.

Villages	Sites	N. Pools/Total Number of Females		N. Positive Pools/Total Number of Females		Minimum Infection Rate	
		2017	2018	2017	2018	2017	2018
Hichria	Dumpsites	7/177	3/52	4/177	3/52	2.25	5.76
	Chenopod fields	1/29	1/16	1/29	1/16	3.44	6.25
Ouled Mohamed	Dumpsites	5/125	5/90	3/125	4/90	2.44	4.44
	Chenopod fields	1/22	1/18	1/22	1/18	4.54	5.55
